# Searching for the Predictors of Response to BoNT-A in Migraine Using Machine Learning Approaches

**DOI:** 10.3390/toxins15060364

**Published:** 2023-05-29

**Authors:** Daniele Martinelli, Maria Magdalena Pocora, Roberto De Icco, Marta Allena, Gloria Vaghi, Grazia Sances, Gloria Castellazzi, Cristina Tassorelli

**Affiliations:** 1 Headache Science and Neurorehabilitation Center, IRCCS Mondino Foundation, 27100 Pavia, Italy; 2 Department of Brain and Behavioral Sciences, University of Pavia, 27100 Pavia, Italy

**Keywords:** migraine, machine learning, onabotulinumtoxinA, predictors of efficacy

## Abstract

OnabotulinumtoxinA (BonT-A) reduces migraine frequency in a considerable portion of patients with migraine. So far, predictive characteristics of response are lacking. Here, we applied machine learning (ML) algorithms to identify clinical characteristics able to predict treatment response. We collected demographic and clinical data of patients with chronic migraine (CM) or high-frequency episodic migraine (HFEM) treated with BoNT-A at our clinic in the last 5 years. Patients received BoNT-A according to the PREEMPT (Phase III Research Evaluating Migraine Prophylaxis Therapy) paradigm and were classified according to the monthly migraine days reduction in the 12 weeks after the fourth BoNT-A cycle, as compared to baseline. Data were used as input features to run ML algorithms. Of the 212 patients enrolled, 35 qualified as excellent responders to BoNT-A administration and 38 as nonresponders. None of the anamnestic characteristics were able to discriminate responders from nonresponders in the CM group. Nevertheless, a pattern of four features (age at onset of migraine, opioid use, anxiety subscore at the hospital anxiety and depression scale (HADS-a) and Migraine Disability Assessment (MIDAS) score correctly predicted response in HFEM. Our findings suggest that routine anamnestic features acquired in real-life settings cannot accurately predict BoNT-A response in migraine and call for a more complex modality of patient profiling.

## 1. Introduction

According to the Global Disease Burden study (GDB), migraine is one of the most disabling neurological conditions in young adults, affecting 14% of the population worldwide [1]. Migraine is classified as chronic (CM) when a headache is present for at least 15 days/month [2]. For when the number of headache days is less than 15 per month, two additional subtypes have been identified for clinical and research purposes: low-to-moderate frequency (up to 6–7 headache days/month) and high-frequency episodic migraine (HFEM) (when the monthly headache days are between 8 and 14) [3]. HFEM is considered a subtype of migraine with a high risk of transformation into CM. Therefore, it appears crucial to focus on preventive treatments that target this condition to reduce migraine frequency and prevent a negative evolution [4].

CM is the most debilitating subtype of migraine [5]. Its treatment currently presents a medical challenge. To date, only onabotulinumtoxinA (BoNT-A), antibodies and small molecules against the Calcitonin Gene-Related Peptide (CGRP) and topiramate are specifically approved for the prophylaxis of CM. BoNT-A inhibits CGRP, P substance and glutamate release as well as transient receptor potential ankyrin 1 (TRPA1) and transient receptor potential vanilloid 1 (TRPV1) translocation, thus switching off peripheral sensitization and, consequently, central sensitization [6]. It was approved by the FDA in 2010 and it is currently administered according to the PREEMPT (Phase III Research Evaluating Migraine Prophylaxis Therapy) protocol, with additional injections in the “follow-the-pain” extension. BoNT-A has also been proved to be effective in reducing the burden of disease in subjects with HFEM when administered according to the PREMPT paradigm in a recent pilot study [7]. A sponsored, larger, multicentric, controlled study is now ongoing to confirm the signal detected in this study (ClinicalTrials.gov Identifier: NCT05028569).

Nevertheless, not all patients adequately respond to BoNT-A injections. Several studies have investigated the possible predictors of treatment responsiveness using clinical, anamnestic, molecular, or imaging features, but they brought only limited evidence [8]. A recent large multicentric study evaluated the rate of excellent responders to BoNT-A and explored the predictors of such responses according to different definitions of efficacy [9]. For example, the factors which were independently associated with an excellent response to BoNT-A, based on the percentage of migraine days reduction, included the presence at baseline of medication overuse and a higher excellent response rate already after the first and the second injection. Females were less likely to present fewer than four monthly headache days.

Unfortunately, most features highlighted as predictors were not replicated in different datasets. Ideally, to be successfully applied in clinical practice, a biomarker should be easily available for analysis (e.g., economically and biologically) and should have a clear cause–effect relationship with the investigated outcome. Overall, there are still no strong single factors correlated with the response to preventive treatment in migraine. Therefore, identifying these features with different approaches is crucial in migraine management, as it could allow targeted therapy, improve patients’ life quality, and diminish healthcare costs, which are all relevant findings when planning treatment strategies in clinical practice.

A machine learning (ML) algorithm is a type of computer program designed to automatically improve its performance by learning from data. Machine learning algorithms use statistical models to identify patterns and relationships in data and use these patterns to make predictions or decisions [10]. There are three types of machine learning algorithms: supervised learning (trained on labelled data), unsupervised learning (trained on unlabeled data), and reinforcement learning (which learns by interacting with the environment). Machine learning algorithms are used in various fields of medicine, including image and speech recognition, natural language processing, and predictive analytics [11]. These machine learning predictive algorithms are being increasingly used in medicine to assist with diagnosis, treatment, and prognosis, as already described in the literature in different areas of application—Alzheimer’s disease, psychiatric disorders, multiple sclerosis, stroke, etc. [12]. When it comes to migraine, some studies applied ML to the setting of correctly classifying primary headache disorders [12,13,14,15,16], whereas only a few of them have focused on predicting treatment response to antibodies against the CGRP [17]. Not only that, but ML could also be applied to extract prognostic information from demographic, clinical and biochemical data to predict the risk to develop medication overuse headache [18].

The primary aim of this single-center, retrospective, real-life study was to detect clinical/anamnestic features able to predict treatment responsiveness in migraine patients undergoing BoNT-A administration for migraine prevention using machine learning algorithms.

## 2. Results

### 2.1. Population Analysed

**Figure 1:**
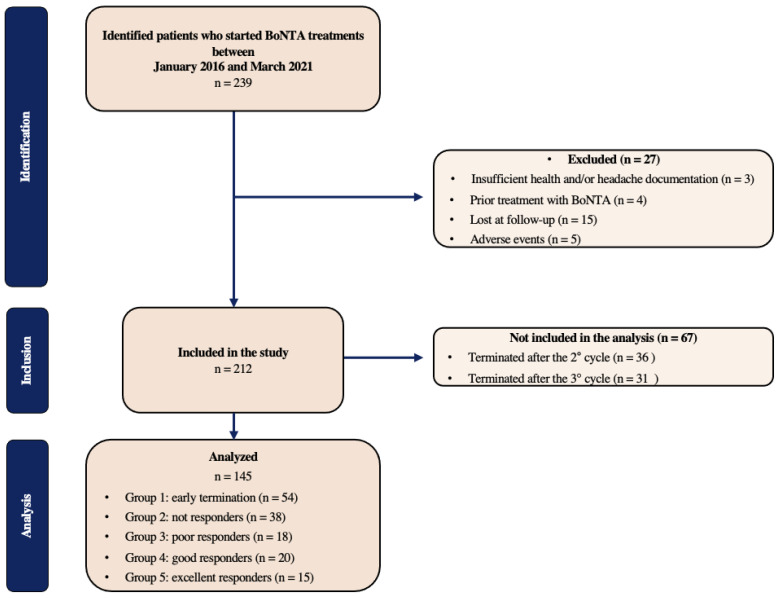
Flowchart of the population included and analyzed in this study.

Two hundred thirty-nine subjects started BoNT-A treatment at IRCCS Mondino Foundation between January 2016 and March 2021. The patients’ disposition is illustrated in Figure 1.

The final analysis was carried out on 91 eligible subjects who completed the four BoNT-A cycles (59 with CM and 32 with HFEM) and on 54 subjects who terminated the treatment after one single cycle (all CM).

A good/excellent response rate (>50% monthly migraine days (MMD) reduction) after 1 year of treatment was observed in 38% of the subjects who completed the four cycles. Demographic and anamnestic features of the population analyzed, divided according to the response rate to BoNT-A, are listed in Table 1 and Table 2.

### 2.2. Machine Learning Analysis

All ML methods applied to the datasets (classified according to the primary or secondary endpoints) failed to discriminate good/excellent responders from nonresponders to BoNT-A administration after the fourth cycle of the treatment using only demographic, anamnestic or clinical features as the input. Table 3 shows the performances using all the different ML approaches applied in the classification comparing groups 2 vs. 4 + 5. The panel of features used for each different classification between groups is listed in Table S1.

When the analysis was extended by including the early terminators (group 1) among the non-responders (groups 1 + 2), the best performance for the primary endpoint with a high classification accuracy of 84.27% (area under the curve (AUC) = 89%) was obtained using 24 baseline features, applying the random forest (RF) algorithm. Table S2 shows the performances and the panel of features resulting from the use of all the different ML approaches applied in the classification comparing groups 1 + 2 vs. groups 4 + 5.

In the subgroup of 32 people with HFEM, BoNT-A reduced MMD at the end of the fourth cycle by 3.68 days (−33.1%, *p* < 0.01) as highlighted in our previous publication [7]. In this subset, RF discriminated responders from nonresponders with a high classification accuracy of 85.71% (AUC = 90.91%) using four baseline features (Table 4). High responsiveness positively correlated with migraine onset age and hospital anxiety and depression score, namely the anxiety subscore (HADS-a). On the other hand, high responsiveness negatively correlated with ongoing opioid use and Migraine Disability Assessment (MIDAS) score at baseline (Table 5).

When forcing the ML algorithms to use the pattern of four features described to validate this model in the cohort of people living with CM, the ML failed to confirm the validity of the model. The performances of the validation protocols are listed in Table S3.

## 3. Discussion

This is the first single-center, real-life retrospective study applying machine learning algorithms to identify factors associated with the efficacy of BoNT-A already at baseline or after the first trimester in a relatively large database of patients.

Clinicians have been seeking biomarkers predictive of BoNT-A effectiveness in migraine for several years now, also searching in many different biological samples, including blood, urine, tissue, and saliva [19]. Acquiring this information is crucial to make the best use of healthcare resources and planning treatments; Table S4 provides an updated summary of the available articles reporting the factors associated with the efficacy of BONT-A in chronic migraine. More specifically, regarding clinical predictors, one of the first features was suggested by Jakubowski et al., who observed that an ocular or imploding phenotype of pain was associated with response to BoNT-A, while exploding pain was not [20]. This finding was later confirmed by some authors [21,22,23], but not by others [24,25]. It appears noteworthy that these studies were conducted with different injection paradigms. Other characteristics were reported as predictive factors of response to BoNT-A in some studies, including unilateral pain, cutaneous allodynia and pericranial muscle tenderness, but there is contrasting evidence as not all studies succeeded in finding these associations [26,27,28,29,30]. Conflicting evidence is also available for the presence/absence of comorbidities (e.g., the presence of depressive symptoms) [31,32] or disease duration [33,34]. CGRP and PTX3 plasmatic levels have also been reported as predictors of responsiveness and/or efficacy [35,36]. Brain structural and functional MRI could also be a tool for assessing response. In a retrospective study, Hubbard et al. demonstrated that BoNT-A responders had increased cortical thickness in the right primary somatosensory cortex, anterior insula left superior temporal gyrus and pars opercularis, compared to nonresponders [37], but it remains unclear whether these findings could be predictive characteristics of the response. Another proposed hallmark of responsiveness was the level of iron accumulated in periaqueductal grey matter, which seemed to correlate with poor response in one study [38].

Overall, despite the mentioned studies, there is currently not enough data to help clinicians make treatment decisions or predict the drug’s effectiveness for a particular patient because the extent of their impact is limited (see Table S4). Moreover, while interesting, testing CGRP or performing an fMRI scan is not yet practical nor economically sustainable in everyday practice.

For this reason, considering the limited prediction potential of a single biomarker, we decided to investigate, with several machine learning algorithms, whether the commonly available clinical information could be combined into a novel panel of features able to predict response to BoNT-A. This approach would provide a predictive tool easily transferable to the real-world setting because the ML-based model is based on anamnestic features that are routinely collected in everyday practice.

The findings obtained with the group of CM patients are partially disappointing because all ML models reached an overall mediocre accuracy but underperformed and lacked specificity. Moreover, due to the low AUC, the algorithms were not able to correctly distinguish one group from the other. A reliable level of discrimination was reached only when a high number of clinical features were considered simultaneously. Precisely, the best performance for the primary endpoint with a high classification accuracy was obtained using 24 features. Considering the requirement of the concomitant presence of all 24 selected features, this condition appears to be impossible to apply in the real-life setting.

This output does not seem related to a limitation of the mathematical approach because the constraint of this analysis lies primarily in the relevance of the information used as an input for the investigation. An example was recently published by Gonzalez-Martinez et al. [17], who used data from a multicentric Spanish database to build an ML model that can predict anti-CGRP response at 6, 9 and 12 months.

More exciting was the output of ML in our subanalysis of the group of HFEM patients, where the random forest algorithm discriminated responders from nonresponders with a high classification accuracy of 85.71% using, altogether, four baseline features: migraine onset age, opioid use, hospital anxiety score, and the disability calculated with the MIDAS score. High responsiveness to BoNT-A treatment after 1 year positively correlated with higher migraine onset age and higher HADS-a score before starting the treatment and negatively correlated with ongoing opioid use as an abortive medication and higher MIDAS score at baseline. These four features represent a panel of easy-to-obtain parameters that, when evaluated together, predict BoNT-A therapy responsiveness in patients with HFEM, even if each feature is not sufficient to fully account for the result.

Unfortunately, this four-feature panel does not predict the response in our cohort of patients with CM. This may be explained by the increased complexity of the pathophysiology when the condition progresses to a more severe pattern, which includes persistent peripheral and central sensitization caused by repetitive and prolonged trigeminal nociceptive activation, and the decrease in endogenous brainstem inhibitory control [39]. This mutated condition is more difficult to treat and is associated with a substantially higher burden of disease, number of comorbidities, and social impact [40].

Machine learning is increasingly being used in the healthcare industry to solve various problems, including clinical decision-making support. Initially, machine learning was mainly used to analyze single-mode data. However, to improve the accuracy of predictions and to simulate the multifaceted nature of clinical decision making, researchers in the field of biomedical machine learning are combining data from different sources to create a more comprehensive dataset [41] on which to apply a multimodal machine learning approach. In the application of BoNT-A to migraine prevention, in order to define a set of features associated with its efficacy, a multimodal approach is, therefore, mandatory to overcome the lack of information when considering only the anamnestic features available. By acquiring and combining data from clinical, radiological, and wet biomarkers, it will probably be possible to build an effective algorithm, even if its applicability will be limited by the cost, reproducibility of the analysis, and technology required to collect/interpret this information. This approach of fusing disparate features, bringing together multiple data sources to capitalize on the unique and complementary information in an algorithmic framework, is aimed at replicating the holistic approach used by clinical experts in their decision-making process. In the near future, medicine will not witness ML overcoming clinical practice, but ML will definitely assist clinicians in their decision-making process [42].

### Limitations

This was a single-center study in a limited, though relatively large, population. Therefore, we were forced to opt for the cross-validation approach to train and test the ML algorithms, which means that the testing records were taken from the same dataset used for training. A second limitation is represented by the retrospective nature of the study, which prevented the collection of some of the features for BoNT-A efficacy that proved interesting with the evolving lines of literature. Information such as the characteristics of the pain (for example, whether it was throbbing or explosive, unilateral or bilateral) and the presence of cranial autonomic symptoms or pericranial muscles tenderness were not collected systematically over the years and, therefore, were not included in the analysis. A prospective study would require their inclusion for an integrated and updated analysis. As a third limitation, the decision to also include in the nonresponder group the patients who stopped the treatment after the first cycle may have resulted in a data selection bias by analyzing as nonresponders patients who might have responded at later times if not discontinued. To control for this, we also applied unsupervised ML algorithms. The last limitation to mention is regarding the sample size, which was unbalanced between the subgroups. The size difference between the early terminator group and the responders might have impacted the statistical power of the ML algorithms. To minimize the potential impact of this, we decided to include both excellent and good responders in the responder group, presenting, therefore, an overall 50% reduction in the MMD, in line with the available literature.

## 4. Conclusions

Overall, ML findings suggest that routine anamnestic features acquired in real-life settings cannot accurately predict the response to BoNT-A treatment in CM patients. A deeper phenotyping of patients’ features, possibly combined with multimodal parameters, is probably required to identify features associated with the response to BoNT-A. In the case of HFEM, ML techniques identified an easy-to-obtain panel of four features that are associated with the response to BoNT-A treatment. This finding is very important and paves the way to tailored therapy in a population of migraine patients who are at high risk of chronification and/or medication overuse.

## 5. Materials and Methods

This was a single-center, observational, retrospective, real-life study conducted at the Headache Science and Rehabilitation Center of the IRCCS Mondino Foundation of Pavia, Italy.

Data from patients with CM or HFEM on preventive treatment with BoNTA between January 2016 and March 2021 were evaluated. Data were previously acquired and anonymized within two different protocols both performed at the IRCCS Mondino Foundation of Pavia (Protocol 1 FM-BOEM authorized by the Pavia local ethical committee on 22 July 2017, reg. n° NCT 04578782 clinicaltrial.gov and Protocol 2 authorized by the Pavia local ethical committee n° 0097925/21). In Italy, studies using retrospective anonymous data from administrative databases, which do not involve direct access for investigators to identification data, do not require further Ethics Committee approval, notification, or patient-informed consent signing.

We selected patients who were started on BoNT-A treatment according to the PREEMPT injection paradigm (155–195 UI in 31–39 injection sites every 12 weeks) for at least one cycle and for whom a prospectively filled headache diary was available for the following 12 months. We excluded patients with insufficient health and/or headache documentation or with significant comorbidities that could have interfered with treatment response. Patients who had already received BoNT-A treatment before the observation period were also excluded. We collected demographic, general health history and headache-specific history. Concomitant medications were collected and classified into with/without potential migraine preventive effect and prescribed/not prescribed for migraine. Information concerning demographics, headache history (especially disease duration at first BoNT-A administration), comorbidities (such as depression, anxiety, low-back pain, hypertension, epilepsy, and sleep apnoea), and previous preventive treatment was also considered. Treatment efficacy was investigated by using self-assessment scales (Migraine Disability Assessment (MIDAS) [43]; Headache Impact Test-6 (HIT6) [44]; Allodynia Symptom Checklist-12 (ASC-12) [45]) after every treatment, in addition to the mere count of migraine/headache days.

### 5.1. Classification

Patients’ response to treatment was used to separate subjects into different groups. They were primarily classified based on their percent-based reduction in monthly migraine days in the 12-week period after the fourth BoNT-A treatment, as compared to a 28-day baseline period (<25%; 25–50%; 50–75%; >75% reduction rate). Patients who terminated treatment early after 1 administration without any effects were profiled as well (early termination (ET)). ET was considered regardless of the clinical indication. The patients terminating the treatment early due to side effects were excluded from the analysis. The patients’ disposition is illustrated in Figure 1.

Groups were labelled as follows based on the outcome at 12 weeks after the first administration cycle or 12 weeks after the 4th administration cycle:

Group 1: early termination, BoNT-A was administered for only 1 cycle due to inefficacy.
Group 2: nonresponders (<25% response after the 4th cycle).
Group 3: poor responders (25–50% response after the 4th cycle).
Group 4: good responders (50–75% response after the 4th cycle).
Group 5: excellent responders (>75% response after the 4th cycle).

The demographic and anamnestic characteristics of the population studied are reported in Table 1 and Table 2, considering this primary working classification. All variables are expressed as mean values ± standard deviation. Secondary classifications were calculated considering the % reduction in abortive medication intake, the % reduction in days in which an abortive medication is required and the MIDAS value % reduction.

### 5.2. Analysis Plan

Considering the entire dataset of people living with migraine, the primary outcome was the prediction of monthly migraine days (MMD) reduction in the 12-week period after the last BoNT-A treatment as compared to baseline. In a real-life monocentric prospective study investigating CM patients treated with BoNT-A, the authors observed that the benefit of the administrations could manifest itself over the canonical 2 cycles, thus suggesting extending treatment forward, to prevent early withdrawal in patients who could become responsive after the 3rd or the 4th cycle [46]. Therefore, we decided to include in the analysis patients with at least 4 subsequent cycles of BoNT-A treatment. The collected data at baseline and after the 1st cycle of BoNT-A were used as input features to run several supervised and unsupervised machine learning algorithms to predict the response to the BoNT-A treatment. The complete ML approaches applied are described in the next sections. Compared to the run-in baseline frequency, a 50% reduction in MMDs after the fourth cycle was considered as a good/excellent response, while a <25% reduction in MMDs was considered a lack of efficacy.

MMDs for each evaluation period were calculated as the mean of the three 4-week segments. We initially compared group 2 (nonresponders) vs. groups 4 + 5 (good and excellent responders). The analysis was subsequently carried out also considering the group of the early terminator as part of the nonresponders’ group (groups 1 + 2 vs. groups 4 + 5).

Secondary endpoints were contemplated as well, and the same analysis plan with the different ML algorithms was carried out, first considering the reduction in abortive medication and, consequently, the number of days when the abortive medications were used. Compared to the run-in baseline condition, a reduction of more than 50% in abortive medication intake or days of drug use after the fourth cycle was considered as a good/excellent response, while a reduction of less than 25% was labelled as a lack of efficacy. As an exploratory endpoint, the reduction in MIDAS value was also evaluated, with the same approaches (classification: responders if >50% reduction after the fourth cycle compared to baseline, nonresponders if <25% reduction).

A final analysis was performed considering the subgroup of HFEM. The primary outcome was the reduction in MMDs in the 12-week period after the last BoNT-A treatment as compared to baseline. Therefore, the collected data were used as input features to run a machine learning algorithm to predict responders (>50% MMD reduction after the fourth administration vs. baseline). The Pearson correlation coefficient was calculated between the resulting features and the percentage of MMD reduction. Finally, the panel of features observed in the HFEM population was applied to the entire CM population to validate its predictive potential.

### 5.3. Machine Learning (ML)

#### 5.3.1. Feature Selection

The feature selection is an essential step for extracting the most informative features for the specific task from a dataset, discarding those which would add only redundant information. In this manuscript, each patient assessment resulted in a long feature vector and, therefore, a very large dataset which could have also led to overfitting issues in the following steps of the analysis. For these reasons, before running any ML code, the entire dataset underwent the application of the ReliefF feature selection algorithm [47], which outputs a ranking of features according to their relevance in determining the class value of the dataset records. After the feature selection step, the data underwent the ML algorithms to build the classification models.

#### 5.3.2. Machine Learning Analysis

In this study, the automatic classification between groups was achieved implementing different ML algorithms (i.e., classifiers): artificial neural network (ANN), support vector machine (SVM), adaptive neuro-fuzzy inference system (ANFIS), random forest and fuzzy c-means clustering (FCM). All the methods were implemented as part of a home-developed [12] tool in Matlab (v. R2018b, The Mathworks, Inc., Natick, MA, USA).

Each ML classifier was run separately to perform model construction and assess validation. For model construction purposes, for each ML classifier, a tuning of the relevant parameters was performed to optimize the setup of the algorithm, therefore maximizing its classification performance. In order to minimize any risk of overfitting, a balanced cross-validation approach (see Section 5.3.3) was adopted and used to train and test each constructed model. For each classifier, we identified the best pool of features to separate patients’ groups with the best classification performance.

##### Artificial Neural Network (ANN)

ANN are ML approaches are inspired by biological neural networks [48]. ANN models are based on connected units (i.e., artificial neurons) that simulate the structure and functionality of brain neurons and their synapses. Among ANN models, the radial basis function network (RBFN) is a feed-forward neural network that uses the radial basis (Gaussian) function as an activation function [10]. Compared to other ANN models, the RBFN special architecture can grant important advantages such as a simpler structure and a faster learning approach.

##### Support Vector Machine (SVM)

Support vector machines (SVMs) are fast and robust classification models that tend to perform very well, even when run on a limited amount of data. SVM algorithms’ objective is to use training data to find the hyperplane in an N-dimensional space (where N is the number of the features) which best separates data from different groups [49]. For the present study, two SVM architectures with a different nonlinear kernel function were used: SVM with the linear kernel (SVM_LIN_) and SVM with radial basis function (RBF) kernel (SVM_RBF_).

##### Adaptive Neuro-Fuzzy Inference System (ANFIS)

ANFIS is a classification approach, in between the ANN methods and the fuzzy logic systems. ANFIS integrates both ANN and fuzzy logic principles and converges the benefits from both methods, offering adaptability, which is characteristic of the ANN backpropagation, and the smoothness that characterizes the fuzzy control interpolation [50]. For this work, we used the ANFIS algorithm included in the fuzzy logic toolbox in Matlab with a Sugeno-type fuzzy inference system (FIS) and Saussian functions as membership functions to specify the fuzzy set.

##### Random Forest (RF)

The random forest approach operates on training data by constructing a forest of decision trees. The classification output is the class predicted by most trees [51]. In this work, the random forest algorithm was implemented in Matlab using the *TreeBagger* function, which uses bootstrap aggregation (i.e., bagging) as an ensemble method to control for overfitting, therefore improving the model generalization.

##### Fuzzy c-Means Clustering (FCM)

Fuzzy c-means clustering (FCM) is a clustering method which allows a data point to belong to more than one cluster [52]. Each data point is assigned to a cluster to some degree which depends on its membership grade. In this work, the FCM approach was implemented in Matlab as part of the homemade classification toolbox.

#### 5.3.3. Cross Validated Accuracy

To improve the classification performances of each ML algorithm, decreasing their variance and, therefore, reducing overfitting issues, a balanced Monte Carlo 10-fold cross-validation (CV) approach using 100 bootstraps was considered [12]. At each run, the CV algorithm splits the original input data into 10 parts, with the dataset classes equally represented, therefore generating 100 new different CV datasets. For each algorithm, for each newly generated CV dataset, nine parts (i.e., nine folds) were used to run the ReliefF feature selection (see Section 5.3.1) and then to train the classifier, while the remaining part (i.e., one fold) was used to test it. For each ML algorithm, the best classification performance, obtained over the 100 bootstraps, and its related model were taken as the final result.

### 5.4. Performance Comparison

In this paper, each ML classifier performed a binary classification to discriminate group A from group B (where A and B are generically used here to address two different groups of patients). For a performance comparison, we calculated classification accuracy, specificity and sensitivity [10]. For each built model, the receiver operating characteristic (ROC) curve was calculated, using its area under the curve (AUC) to compare the different classifiers’ performance [53].

Two hundred thirty-nine subjects started BoNT-A treatment at IRCCS Mondino Foundation between January 2016 and March 2021. Twenty-seven patients were excluded because it was not possible to track medical records (n = 3) or those had already used BoNTA (n = 4). Fifteen were lost at follow up and four presented an adverse event, because of which it was decided not to proceed with the treatment. Of the remaining 212, 32 were affected by HFEM, and the remaining 204 suffered from CM. The analysis was carried out on 145 eligible subjects, of whom 91 completed the 4 BoNT-A cycles, while 54 terminated the treatment early after 1 single cycle. Sixty-seven subjects who decided to terminate the treatment after the second or third cycle were not included in the analysis.

## Figures and Tables

**Table 1:** Demographic characteristics.

	Group 1	Group 2	Group 3	Group 4	Group 5
	Early Termination	<25% Response Rate	25–50% Response Rate	50–75% Response Rate	>75% Response Rate
Gender	48F. 6M	30F. 8M	13F. 5M	17F. 3M	13F. 2M
Disease duration, years	31.8 ± 15.4	34.7 ± 14.0	34.9 ± 11.8	31.45 ± 17.7	32.7 ± 17.9
Preventive drugs previously tried, n.	3.9 ± 1.3	3.3 ± 2.1	2.9 ± 1.5	2.5 ± 1.8	2.5 ± 1.45
Comorbidity: Mood disorder, n (%)	28 (51.8%)	20 (52.6%)	6 (33.3%)	10 (50.0%)	6 (40.0%)
Anxiety, n (%)	33 (61.1%)	23 (60.5%)	12 (66.7%)	9 (45.0%)	8 (53.3%)
Low back pain, n (%)	4 (7.4%)	9 (23.7%)	3 (16.7%)	3 (15.0%)	4 (26.7%)
Hypertension, n (%)	10 (18.5%)	7 (18.4%)	4 (22.2%)	5 (25.0%)	4 (26.7%)
Sleep apnea, n (%)	0	0	0	0	1 (6.7%)
Epilepsy, n (%)	1 (1.8%)	2 (5.3%)	0	1 (5.0%)	0
MOH before, n (%)	11 (20.4%)	15 (39.5%)	6 (33.3%)	5 (25.0%)	3 (20.0%)
MOH now, n (%)	37 (68.5%)	12 (31.6%)	6 (33.3%)	8 (40.0%)	8 (53.3%)
Opioid use before, n (%)	11 (20.4%)	0	2 (11.1%)	1 (5.0%)	0
Opioid use now, n (%)	11 (20.4%)	7 (18.4%)	4 (22.2%)	3 (15.0%)	1 (6.7%)
CT + M, n (%)	18 (33.3%)	23 (60.5%)	8 (44.4%)	9 (45.0%)	6 (40.0%)
CT + O, n (%)	33 (61.1%)	23 (60.5%)	11 (61.1%)	10 (50.0%)	11 (73.3%)

Table: Groups were labelled as follows: Group 1: early termination (after 1 BoNTA administration); Group 2: nonresponders (<25% response after the 4th cycle); Group 3: poor responders (25–50% response after the 4th cycle); Group 4: good responders (50–75% response after the 4th cycle); Group 5: excellent responders (>75% response after the 4th cycle). All variables are expressed as mean value, ±standard deviation. Legend: F = female; M = male; n = number; MOH = medication overuse headache; CT + M = concomitant treatment prescribed for migraine; CT + O = concomitant treatment prescribed for other indication.

**Table 2:** Headache anamnestic characteristics.

	Group 1 (n. 54)		Group 2 (n. 38)		Group 3 (n. 18)		Group 4 (n. 20)		Group 5 (n. 15)	
	Baseline	1st Trimester	Baseline	4th Trimester	Baseline	4th Trimester	Baseline	4th Trimester	Baseline	4th Trimester
Migraine days, n	24.4 ± 5.1	22 ± 6.4	18.9 ± 7.5	20.6 ± 11.1	16.2 ± 6.6	10.6 ± 4.3	17.65 ± 6.95	7.4 ± 2.6	22.7 ± 7.1	4.2 ± 2.1
Headache days, n	24.1 ± 5.4	21.9 ± 6.3	19.8 ± 7.5	20.8 ± 13.8	17.4 ± 6.8	10.7 ± 4.3	17.95 ± 6.7	10.3 ± 7.7	22.5 ± 7.1	7.2 ± 8.6
Abortive medication, n	26.8 ± 31.3	16.8 ± 13.5	20.6 ± 21.5	19.9 ± 21.4	15.8 ± 8.3	11.3 ± 6.95	18.85 ± 16.25	7.9 ± 4.6	26.05 ± 16.5	3.0 ± 1.8
MIDAS, n	80.1 ± 51.8	74.9 ± 51.0	63.7 ± 52.6	43.0 ± 32.1	42.2 ± 27.0	31.6 ± 30.6	46.3 ± 43.1	21.1 ± 25.5	60.4 ± 47.6	5.7 ± 8.4
HIT6, n	66.8 ± 5.6	64.9 ± 5.1	66.1 ± 6.0	63.6 ± 4.9	64.75 ± 3.3	60.25 ± 7.2	66.5 ± 5.8	60.75 ± 6.6	66.8 ± 7.9	57 ± 10.3
ASC-12, n	6.2 ± 4.4	6.2 ± 4.2	6.1 ± 3.7	5.5 ± 3.95	7.0 ± 3.9	6.8 ± 4.15	4.6 ± 4.1	3.4 ± 2.6	3.9 ± 2.8	2.3 ± 5.1

Table: Groups were labelled as follows: Group 1: early termination (after 1 BoNTA administration); Group 2: nonresponders (<25% response after the 4th cycle); Group 3: poor responders (25–50% response after the 4th cycle); Group 4: good responders (50–75% response after the 4th cycle); Group 5: excellent responders (>75% response after the 4th cycle). All variables are expressed as mean value, ±standard deviation. Legend: n = number; MIDAS = Migraine Disability Assessment; HIT6 = Headache Impact Test-6; ASC-12 = Allodynia Symptom Checklist-12.

**Table 3:** Results of the different machine learning methods to classify responders vs. nonresponders after the fourth cycle.

Machine Learning Methods	ACC (%)	Sens (%)	Spec (%)	AUC (%)	N Features
**PRIMARY ENDPOINT—migraine days reduction**					
Random forest	100	100	100	16,67	12
SVM (linear kernel)	76.67	63.33	90	23.33	3
SVM (RBF kernel)	76.67	70	83.33	23.33	2
ANFIS (aNN)	50	100	0	20	1
MLP (aNN)	85	90	80	15	2
Fuzzy clustering (unsup. ML)	45	90	0	86.67	2
SECONDARY ENDPOINT—abortive medication intake reduction					
Random forest	100	100	100	33	8
SVM (linear kernel)	83.33	76,67	90	16.67	4
SVM (RBF kernel)	83.33	83033	83.33	16.67	5
ANFIS (aNN)	50	100	0	21.67	1
MLP (aNN)	81.67	83.33	80	18.33	3
Fuzzy clustering (unsup. ML)	48.33	96.67	0	56.67	4
SECONDARY ENDPOINT—reduction in days in which an abortive medication is required					
Random forest	100	100	100	12.12	12
SVM (linear kernel)	88.33	76.67	100	11.67	5
SVM (RBF kernel)	75	90	60	25	3
ANFIS (aNN) *	-	-	-	-	-
MLP (aNN)	88.33	80	86.67	16.67	4
Fuzzy clustering (unsup. ML)	50	100	0	50	1
EXPLORATORY ENDPOINT—MIDAS reduction					
Random forest	100	100	100	27.27	2
SVM (linear kernel)	85	80	90	15	3
SVM (RBF kernel)	67.5	55	80	32.5	1
ANFIS (aNN) *	-	-	-	-	-
MLP (aNN)	85	85	85	15	2
Fuzzy clustering (unsup. ML)	47.5	95	0	57.5	1

Table: The population is composed of only those patients who completed 1 year of treatment. The comparison is performed between group 2 vs. 4 + 5. Legend: accuracy (ACC); sensibility (Sens); specificity (Spec); area under the curve (AUC); the number of features used for classification (N); suppor vector machine (SVM); RBF kernel); ANFIS (aNN); MLP (aNN); unsupervised (unsup.); * performance failure in predicting the outcome. Data are expressed as percentage (%).

**Table 4:** Responsiveness prediction in people living with high-frequency episodic migraine with a panel of 4 baseline features.

Statistics	%; [95% C + I]
Accuracy	85.71% [0.66–0.96]
Sensibility	94.12% [0.77–1.00]
Specificity	72.12% [0.52–0.88]
Precision	84.21 % [0.65–0.95]
f-measure	88.89% [0.70–0.98]
Area under the curve (AUC)	90.91% [0.73–0.99]

Table: Summary of the random forest performance scores using the 4 selected features at baseline as a discriminant pattern in the subset population of people living with high-frequency episodic migraine. The data are expressed as percentage (%); 95% CI = confidence interval [lower bound; upper bound].

**Table 5:** Correlations in the HFEM population.

Features	Pearson Correlation	*p*-Value
Migraine age onset	+0.488	0.009
MIDAS	−0.245	0.209
HADS-A score	+0.418	0.027
Ongoing opioid use as an abortive medication	−0.509	0.006

Table: Summary table reporting the relevant features resulting from the RF algorithm and their individual Pearson correlation coefficients, related to the clinical response size (% reduction in MMD after the fourth cycle) in the subset of patients suffering from high-frequency episodic migraine.

## Data Availability

Data used for this study are available from the corresponding author upon request.

## Supplementary Materials

The following supporting information can be downloaded at: https://www.mdpi.com/article/10.3390/toxins15060364/s1, Table S1: List of the features evaluated with each different ML algorithm. Results of the different machine learning (ML) approaches applied to primary, secondary, and exploratory endpoints. The population is composed of only those patients who completed 1 year of treatment. The comparison is performed between groups 2 vs. 4 + 5. Legend: accuracy (ACC); sensibility (Sens); specificity (Spec); area under the curve (AUC); the number of features used for classification (N); support vector machine (SVM); RBF kernel; ANFIS (aNN); MLP (aNN); unsupervised (unsup.) * performance failure in predicting the outcome. Data are expressed as percentage (%). Table S2: Results, responders vs. nonresponders or early terminator. Results of the different machine learning (ML) approaches applied to primary, secondary, and exploratory endpoints. The population is composed of those patients who completed 1 year of treatment as well as the early terminator who lacked efficacy after 1 cycle and decided to stop the treatment. The comparison is performed between groups 1 + 2 vs. 4 + 5. Legend: accuracy (ACC); sensibility (Sens); specificity (Spec); area under the curve (AUC); the number of features used for classification (N); support vector machine (SVM); RBF kernel; ANFIS (aNN); MLP (aNN); unsupervised (unsup.). Table S3: Validation, in people living with chronic migraine, of the 4-feature panel obtained with the population of people living with high-frequency episodic migraine. The comparison is performed between groups 1 + 2 vs. 4 + 5. Legend: accuracy (ACC); sensibility (Sens); specificity (Spec); area under the curve (AUC); the number of features used for classification (N); support vector machine (SVM); RBF kernel; ANFIS (aNN); MLP (aNN); unsupervised (unsup.). Table S4: Summary of the available literature regarding BonT-A efficacy and predictors.

## References

[1] Stovner L.J., Hagen K., Linde M., Steiner T.J. (2022). The Global Prevalence of Headache: An Update, with Analysis of the Influences of Methodological Factors on Prevalence Estimates. J. Headache Pain.

[2] Olesen J. (2018). Headache Classification Committee of the International Headache Society (IHS). The International Classification of Headache Disorders.

[3] Serrano D., Lipton R.B., Scher A.I., Reed M.L., Stewart W.F., Adams A.M., Buse D.C. (2017). Fluctuations in Episodic and Chronic Migraine Status over the Course of 1 Year: Implications for Diagnosis, Treatment and Clinical Trial Design. J. Headache Pain.

[4] Lipton R.B. (2009). Tracing Transformation: Chronic Migraine Classification, Progression, and Epidemiology. Neurology.

[5] Torres-Ferrús M., Quintana M., Fernandez-Morales J., Alvarez-Sabin J., Pozo-Rosich P. (2017). When Does Chronic Migraine Strike? A Clinical Comparison of Migraine According to the Headache Days Suffered per Month. Cephalalgia.

[6] Martinelli D., Arceri S., Tronconi L., Tassorelli C. (2020). Chronic Migraine and Botulinum Toxin Type A: Where Do Paths Cross?. Toxicon.

[7] Martinelli D., Arceri S., De Icco R., Allena M., Guaschino E., Ghiotto N., Bitetto V., Castellazzi G., Cosentino G., Sances G. (2022). BoNT-A Efficacy in High Frequency Migraine: An Open Label, Single Arm, Exploratory Study Applying the PREEMPT Paradigm. Cephalalgia.

[8] Ray J.C., Hutton E.J., Matharu M. (2021). Onabotulinumtoxina in Migraine: A Review of the Literature and Factors Associated with Efficacy. J. Clin. Med..

[9] Ornello R., Baraldi C., Ahmed F., Negro A., Miscio A.M., Santoro A., Alpuente A., Russo A., Silvestro M., Cevoli S. (2022). Excellent Response to OnabotulinumtoxinA: Different Definitions, Different Predictors. Int. J. Environ. Res. Public Health.

[10] Christopher M. (2006). Bishop Pattern Recognition and Machine Learning.

[11] Sidey-Gibbons J.A.M., Sidey-Gibbons C.J. (2019). Machine Learning in Medicine: A Practical Introduction. BMC Med. Res. Methodol..

[12] Castellazzi G., Cuzzoni M.G., Cotta Ramusino M., Martinelli D., Denaro F., Ricciardi A., Vitali P., Anzalone N., Bernini S., Palesi F. (2020). A Machine Learning Approach for the Differential Diagnosis of Alzheimer and Vascular Dementia Fed by MRI Selected Features. Front. Neuroinform..

[13] Ferroni P., Zanzotto F.M., Scarpato N., Spila A., Fofi L., Egeo G., Rullo A., Palmirotta R., Barbanti P., Guadagni F. (2020). Machine Learning Approach to Predict Medication Overuse in Migraine Patients. Comput. Struct. Biotechnol. J..

[14] Garcia-Chimeno Y., Garcia-Zapirain B., Gomez-Beldarrain M., Fernandez-Ruanova B., Garcia-Monco J.C. (2017). Automatic Migraine Classification via Feature Selection Committee and Machine Learning Techniques over Imaging and Questionnaire Data. BMC Med. Inform. Decis. Mak..

[15] Messina R., Filippi M. (2020). What We Gain From Machine Learning Studies in Headache Patients. Front. Neurol..

[16] Rocca M.A., Harrer J.U., Filippi M. (2020). Are Machine Learning Approaches the Future to Study Patients with Migraine?. Neurology.

[17] Gonzalez-Martinez A., Pagán J., Sanz-García A., García-Azorín D., Rodríguez-Vico J.S., Jaimes A., García A.G., de Terán J.D., González-García N., Quintas S. (2022). Machine-Learning-Based Approach for Predicting Response to Anti-Calcitonin Gene-Related Peptide (CGRP) Receptor or Ligand Antibody Treatment in Patients with Migraine: A Multicenter Spanish Study. Eur. J. Neurol..

[18] Parrales Bravo F., Del Barrio García A.A., Gallego M.M., Gago Veiga A.B., Ruiz M., Guerrero Peral A., Ayala J.L. (2019). Prediction of Patient’s Response to OnabotulinumtoxinA Treatment for Migraine. Heliyon.

[19] Demartini C., Francavilla M., Zanaboni A.M., Facchetti S., De Icco R., Martinelli D., Allena M., Greco R., Tassorelli C. (2023). Biomarkers of Migraine: An Integrated Evaluation of Preclinical and Clinical Findings. Int. J. Mol. Sci..

[20] Jakubowski M., Mcallister P.J., Bajwa Z.H., Ward T.N., Smith P., Burstein R. (2006). Exploding vs. Imploding Headache in Migraine Prophylaxis with Botulinum Toxin A. Pain.

[21] Kim C.C., Bogart M.M., Wee S.A., Burstein R., Arndt K.A., Dover J.S. (2010). Predicting Migraine Responsiveness to Botulinum Toxin Type A Injections. Arch. Dermatol..

[22] Grogan P.M., Alvarez M.V., Jones L. (2013). Headache Direction and Aura Predict Migraine Responsiveness to Rimabotulinumtoxin B. Headache.

[23] Burstein R., Dodick D., Silberstein S. (2009). Migraine Prophylaxis with Botulinum Toxin A Is Associated with Perception of Headache. Toxicon.

[24] Lin K.H., Chen S.P., Fuh J.L., Wang Y.F., Wang S.J. (2014). Efficacy, Safety, and Predictors of Response to Botulinum Toxin Type A in Refractory Chronic Migraine: A Retrospective Study. J. Chin. Med. Assoc..

[25] Pagola I., Esteve-Belloch P., Palma J.A., Luquin M.R., Riverol M., Martínez-Vila E., Sieira P.I. (2014). Predictive Factors of the Response to Treatment with Onabotulinumtoxina in Refractory Migraine. Rev. Neurol..

[26] De Tommaso M., Brighina F., Delussi M. (2019). Effects of Botulinum Toxin A on Allodynia in Chronic Migraine: An Observational Open-Label Two-Year Study. Eur. Neurol..

[27] Mathew N.T., Kailasam J., Meadors L. (2008). Predictors of Response to Botulinum Toxin Type A (BoNTA) in Chronic Daily Headache. Headache J. Head Face Pain.

[28] Young W.B., Ivan Lopez J., Rothrock J.F., Orejudos A., Manack Adams A., Lipton R.B., Blumenfeld A.M. (2019). Effects of OnabotulinumtoxinA Treatment in Patients with and without Allodynia: Results of the COMPEL Study. J. Headache Pain.

[29] Sandrini G., Perrotta A., Tassorelli C., Torelli P., Brighina F., Sances G., Nappi G. (2011). Botulinum Toxin Type-A in the Prophylactic Treatment of Medication-Overuse Headache: A Multicenter, Double-Blind, Randomized, Placebo-Controlled, Parallel Group Study. J. Headache Pain.

[30] Lovati C., Giani L., Mariotti Dalessandro C., Tabaee Damavandi P., Mariani C., Pantoni L. (2018). May Migraine Attack Response to Triptans Be a Predictor of the Efficacy of Onabotulinum Toxin-A Prophylaxis?. Neurol. Sci..

[31] Eren O.E., Gaul C., Peikert A., Gendolla A., Ruscheweyh R., Straube A. (2020). Triptan Efficacy Does Not Predict OnabotulinumtoxinA Efficacy but Improves with OnabotulinumtoxinA Response in Chronic Migraine Patients. Sci. Rep..

[32] Di Cola F.S., Caratozzolo S., Liberini P., Rao R., Padovani A. (2019). Response Predictors in Chronic Migraine: Medication Overuse and Depressive Symptoms Negatively Impact Onabotulinumtoxin-A Treatment. Front. Neurol..

[33] Domínguez C., Pozo-Rosich P., Torres-Ferrús M., Hernández-Beltrán N., Jurado-Cobo C., González-Oria C., Santos S., Monzón M.J., Latorre G., Álvaro L.C. (2017). OnabotulinumtoxinA in Chronic Migraine: Predictors of Response. A Prospective Multicentre Descriptive Study. Eur. J. Neurol..

[34] Eross E.J., Gladstone J.P., Lewis S., Rogers R., Dodick D.W. (2005). Duration of Migraine Is a Predictor for Response to Botulinum Toxin Type A. Headache.

[35] Cernuda-Morollón E., Ramón C., Martínez-Camblor P., Serrano-Pertierra E., Larrosa D., Pascual J. (2015). OnabotulinumtoxinA Decreases Interictal CGRP Plasma Levels in Patients with Chronic Migraine. Pain.

[36] Domínguez C., Vieites-Prado A., Pérez-Mato M., Sobrino T., Rodríguez-Osorio X., López A., Campos F., Martínez F., Castillo J., Leira R. (2018). CGRP and PTX3 as Predictors of Efficacy of Onabotulinumtoxin Type A in Chronic Migraine: An Observational Study. Headache J. Head Face Pain.

[37] Hubbard C.S., Becerra L., Smith J.H., DeLange J.M., Smith R.M., Black D.F., Welker K.M., Burstein R., Cutrer F.M., Borsook D. (2016). Brain Changes in Responders vs. Non-Responders in Chronic Migraine: Markers of Disease Reversal. Front. Hum. Neurosci..

[38] Vivero C.D., Leira Y., Piñeiro M.S., Rodríguez-Osorio X., Ramos-Cabrer P., Martín C.V., Sobrino T., Campos F., Castillo J., Leira R. (2020). Iron Deposits in Periaqueductal Gray Matter Are Associated with Poor Response to Onabotulinumtoxina in Chronic Migraine. Toxins.

[39] Rattanawong W., Rapoport A., Srikiatkhachorn A. (2022). Neurobiology of Migraine Progression. Neurobiol. Pain.

[40] Mungoven T.J., Henderson L.A., Meylakh N. (2021). Chronic Migraine Pathophysiology and Treatment: A Review of Current Perspectives. Front. Pain Res..

[41] Kline A., Wang H., Li Y., Dennis S., Hutch M., Xu Z., Wang F., Cheng F., Luo Y. (2022). Multimodal Machine Learning in Precision Health: A Scoping Review. NPJ Digit. Med..

[42] Noorbakhsh-Sabet N., Zand R., Zhang Y., Abedi V. (2019). Artificial Intelligence Transforms the Future of Health Care. Am. J. Med..

[43] D’Amico D., Mosconi P., Genco S., Usai S., Prudenzano A.M.P., Grazzi L., Leone M., Puca F.M., Bussone G. (2001). The Migraine Disability Assessment (MIDAS) Questionnaire: Translation and Reliability of the Italian Version. Cephalalgia.

[44] Yang M., Rendas-Baum R., Varon S.F., Kosinski M. (2011). Validation of the Headache Impact Test (HIT-6^TM^) across Episodic and Chronic Migraine. Cephalalgia.

[45] Florencio L.L., Chaves T.C., Branisso L.B., Gonçalves M.C., Dach F., Speciali J.G., Bigal M.E., Bevilaqua-Grossi D. (2012). 12 Item Allodynia Symptom Checklist/Brasil: Cross-Cultural Adaptation, Internal Consistency and Reproducibility. Arq. Neuropsiquiatr..

[46] Sarchielli P., Romoli M., Corbelli I., Bernetti L., Verzina A., Brahimi E., Eusebi P., Caproni S., Calabresi P. (2017). Stopping Onabotulinum Treatment after the First Two Cycles Might Not Be Justified: Results of a Real-Life Monocentric Prospective Study in Chronic Migraine. Front. Neurol..

[47] Kononenko I., Šimec E., Robnik-Šikonja M. (1997). Overcoming the Myopia of Inductive Learning Algorithms with RELIEFF. Appl. Intell..

[48] Simon H. (1998). Neural Networks: A Comprehensive Foundation: A Comprehensive Foundation.

[49] Steinwart I., Christmann A. (2008). Support Vector Machines.

[50] Zadeh L.A. (1978). Fuzzy Sets as a Basis for a Theory of Possibility. Fuzzy Sets Syst..

[51] Breiman L. (2001). Random Forests. Mach. Learn..

[52] Dunn J.C. (1973). A Fuzzy Relative of the ISODATA Process and Its Use in Detecting Compact Well-Separated Clusters. J. Cybern..

[53] Hanley J.A., McNeil B.J. (1982). The Meaning and Use of the Area under a Receiver Operating Characteristic (ROC) Curve1. Radiology.

