# Supplementary material for: Searching for the Predictors of Response to BoNT-A in Migraine Using Machine Learning Approaches
Source: Toxins (Basel). 2023 May 29;15(6):364. doi: 10.3390/toxins15060364 (PMC10303214; doi:10.3390/toxins15060364)
Supplement: Supplementary file 1 [file toxins-15-00364-s001.zip › tableS4_literature.pdf]

Study (First author, year, (DOI))	Population (n)	Diagnosis	Treatment	Follow-up (months)	Definition of response	Significant predictors observed	Statistical relevance of the predictors observed	Non significant predictors
Ornello, 2022 (doi.org/10.3390/ijerph191710975)	n= 2879	CM	155-195 UI	9	patients with a ≥75% decrease in monthly headache days (percent-based excellent responders)	Medication overuse at baseline; medication consumption at the baseline; an excellent response during the first and the second cycle of BoNT-A	Compared with non-responders, percent-based excellent responders displayed a higher prevalence of medication overuse (71.7% vs. 63.8%, *p* = 0.001) and a higher medication consumption at the baseline (20.97 ± 9.45 vs. 18.10 ± 9.95, *p* < 0.001); moreover, percent-based excellent responders at the 9th month displayed a higher proportion of excellent responders even at the 3rd (52.85% vs. 47.15%, *p* < 0.001) and the 6th months (77.83% vs. 22.17%, *p* < 0.001). At the multivariate analysis, the presence of MO at the baseline and an excellent response during the first and the second BoNT-A cycles were significantly associated with percent-based excellent response during the third BT-A cycle	
					<4 MMD (frequency-based excellent responders)	Sex: female population	72% vs 80% p=0.041	

Eren, 2020 (10.1038/s41598-020-68149-1)	n=49	CM	155 UI	3	≥30% fewer MMD	None	N/A	Triptan efficacy
Domínguez Vivero, 2020 (doi.org/10.3390/toxins12080479)	n=62	CM	155 UI	3	≥50% fewer headaches	Age, CGRP levels (> 50 ng/mL), Pentraxin 3 (PTX3) levels (>1000 pg/ml), iron deposition in GP and PAG	mean age difference = 12.2; 95% confidence interval (CI): 5.4–18.9, p = 0.001 CGRP mean difference = 805.0; 95% CI: 37.9–1572.1 µL, p = 0.040 PTX3 mean difference = 69.8; 95% CI: 31.0–108.6 µL, p = 0.008;	Gender, BMI, smoking, headache phenotype, allodynia, presence or location of WML
Moreno-Mayordomo, 2019 (10.1186/s10194-019-0989-9.)	n=156	CM	155 UI	6	≥50% fewer MMD	Polymorphisms : -CALCA rs3781719 - TRPV1 rs222749	p = 0.007, OR = 3.11 (1.33-7.26) and (p = 0.013, OR = 3.29 (1.28-8.43) respectively	23 other SNPs
Schiano di Cola, 2019 (10.3389/fneur.2019.00678. eCollection 2019)	n=84	CM	155-195 UI	12	≥50% fewer headaches	Depressive symptoms, MOH	N/A	N/A
de Tommaso, 2019 (10.1159/000499764.)	n=120	CM	155-195 UI	24	≥50% fewer headaches	Less severe allodynia	relationship between allodynia severity at T0 and reduced headache frequency change at T1 (r = 0.22) and T2 (r = 0.37)	N/A
Young, 2019 (doi.org/10.1186/s10194-018-0952-1)	n=175	CM	155 UI	27	Mean reduction MHD	Absence allodynia	Mean reduction in MMD was significantly greater in patients without allodynia (P = 0.044 between-subgroup comparison)	N/A
Domínguez, 2018 (10.1111/head.13211.)	n=62	CM	155 UI	6	≥50% fewer headaches	CGRP levels, PTX3 levels	PTX3 serum levels (1455.4 ± 487.5 pg/mL versus 720.3 ± 334.1 pg/mL, P < .0001) and CGRP serum levels (133.1 ± 86.6 ng/mL versus 58.2 ± 91.7 ng/mL, P = .004) were significantly higher in responders than nonresponders.	TNF-a, IL-10, IL-6, hs-CRP, cFn, S100, NSE,

Domínguez, 2018 (doi.org/10.1111/ene.13523)	n=725	CM	155 UI	12	≥50% fewer headaches	CM duration, Unilateral pain, combined symptomatic treatment, fewer days of disability, milder headache	Correlation analysis of data showed that patients with a shorter duration of disease (r=-0.108; p=0.004), those who had less days of disability per month (r=-0.144; p=0.001), and a milder headache (r=-0.098; p=0.008) had more chances of responding to treatment with OnabotulinumtoxinA. Conversely, we found a positive correlation between unilaterality of pain and effectiveness (r=0.093; p=0.012). The ROC analysis for duration of disease showed an area under the curve of 0.441 (CI 95% 0.388-0.493, p=0.025), suggesting that a value below 12 months of duration of CM predicts effectiveness with a sensitivity of 72% and a specificity of 78%.	Gender, Migraine with aura, depression, fibromyalgia
Lovati, 2018 (10.1007/s10072-018-3388-0.)	n=44	CM	170-195 UI	12	≥50% fewer MMD	Triptan response	$\chi 2$ test (3 lines × 3 columns), a significant difference between groups was observed (p = 0.016).	N/A
Hubbard, 2016 (doi.org/10.3389/fnhum.2016.00497)	n=23	CM	150 UI	6	≥50% fewer MHD and now EM	Cortical thickness at right SI and aINS, left ParsOp and STG	anti-correlations between the SI seed and lateral occipital (LOC) and dorsomedial prefrontal cortices (DMPFC) in responders, whereas non-responders	N/A

							showed increased connectivity between the ParsOp seed and LOC	
Lee, 2016 (doi.org/10.1016/j.jns.2016.01.054)	n=70	CM	155 UI	1,5	≥50% fewer MMD, ≥50%fewer abortive Rx OR ≥50% fewer mod-severe headache	Disease Duration, MCA/ICA ratio	Longer disease duration was associated with poor treatment outcome ($p = 0.019$). The ratio of the mean velocity of middle cerebral artery to that of ipsilateral internal carotid artery (MCA/ICA index) was significantly higher in responders than non-responders ($p< 0.027$).	Age, gender, BMI, concurrent prophylactic medication, MA vs. MoA, psychiatric comorbidity, smoking
Cernuda-Morollón, 2015 (10.1097/j.pain.0000000000000119)	n=83	CM	155-195 UI	6	≥50% fewer headaches AND ≥50% reduced VAS	CGRP (76.85 pg/mL vs. 50.45 pg/mL), VIP	Pre-treatment CGRP levels in responders (76.85 pg/mL) were significantly higher than those seen in nonresponders (50.45 pg/mL; $P < 0.001$).	Clinical features, age, duration of CM, comorbidity, obesity, prophylactic medication

eTable 4. *Possible predictive factors for the efficacy of BoNT-A treatment following PREEMPT paradigm, adapted from Ray et al 2021 (https://doi.org/10.3390/jcm10132898)*

Legend:CM chronic migraine; EM episodic migraine; UI international unit, MMD migraine monthly days, VAS Visual assessment score, MCA middle cerebral artery, ICA internal carotid artery, MOH medication overuse headache
